# Enhanced Noise-Resilient Pressure Mat System Based on Hyperdimensional Computing

**DOI:** 10.3390/s24031014

**Published:** 2024-02-04

**Authors:** Fatemeh Asgarinejad, Xiaofan Yu, Danlin Jiang, Justin Morris, Tajana Rosing, Baris Aksanli

**Affiliations:** 1 Electrical and Computer Engineering, University of California San Diego, La Jolla, CA 92093, USA; fasgarinejad@ucsd.edu (F.A.); x1yu@ucsd.edu (X.Y.); d1jiang@ucsd.edu (D.J.);; 2 Electrical and Computer Engineering, San Diego State University, San Diego, CA 92182, USA; 3 Computer Science and Information Systems, California State University San Marcos, San Marcos, CA 92096, USA; justinmorris@csusm.edu

**Keywords:** pressure sensing, hyperdimensional computing, human activity recognition

## Abstract

Traditional systems for indoor pressure sensing and human activity recognition (HAR) rely on costly, high-resolution mats and computationally intensive neural network-based (NN-based) models that are prone to noise. In contrast, we design a cost-effective and noise-resilient pressure mat system for HAR, leveraging Velostat for intelligent pressure sensing and a novel hyperdimensional computing (HDC) classifier that is lightweight and highly noise resilient. To measure the performance of our system, we collected two datasets, capturing the static and continuous nature of human movements. Our HDC-based classification algorithm shows an accuracy of 93.19%, improving the accuracy by 9.47% over state-of-the-art CNNs, along with an 85% reduction in energy consumption. We propose a new HDC noise-resilient algorithm and analyze the performance of our proposed method in the presence of three different kinds of noise, including memory and communication, input, and sensor noise. Our system is more resilient across all three noise types. Specifically, in the presence of Gaussian noise, we achieve an accuracy of 92.15% (97.51% for static data), representing a 13.19% (8.77%) improvement compared to state-of-the-art CNNs.

## 1. Introduction

Smart homes utilize cost-effective and user-friendly motion and vibration sensors [1,2,3] among others. These sensors facilitate continuous monitoring, playing an integral role in human activity recognition within the home environment. Recent advancements in machine learning algorithms have enabled the detection and classification of more sophisticated indoor activities [1,2,4], further enhancing the capabilities of smart home systems [1,2,4]. In the context of smart home applications, pressure sensing mats are an important type of sensor [5,6,7]. Pressure mats are used for elderly care [5,6,7], health monitoring [7,8], security [8,9], daily activity monitoring [5,9,10,11,12,13,14], analyzing walking gait in ASD children [15], and recognizing sleep postures to optimize sleep positions for improved sleep quality and overall health [16].

Related works on human activity recognition using pressure mats cover a range of design areas, encompassing hardware design, algorithm design, and system design, where the latter includes both hardware and algorithmic aspects. Notably, Chen et al. introduced a novel contactless sleep-monitoring IoT system with RFID-tagged bed sheets and a Raspberry Pi 4 Model B, addressing the limitations of conventional sleep posture monitoring based on the Random Forest method [17]. The work by Tang et al. [7] used a convolutional neural network (CNN) for posture recognition and provided real-time feedback, posture diaries, and fall alerts through a smartphone application, offering an advanced and accessible solution compared to intrusive and expensive alternatives for clinical sleep monitoring. Stern et al. proposed 2D and 3D CNNs for in-bed posture monitoring and achieved high accuracy for detecting different sleep positions in an open-access dataset [18]. The study addressed dataset imbalance using strategies such as downsampling. In [19], Clever et al. introduced PressurePose, a synthetic dataset featuring a large pressure images dataset with 3D human poses and shapes, created through a physics-based simulation method. The accompanying PressureNet is a deep learning model which, with the aid of a pressure map reconstruction (PMR) network, estimates human pose and shape from pressure images, exhibiting robust performance with real data for diverse poses.

On the algorithm side, convolutional neural networks (CNNs) have demonstrated outstanding performance in various activity recognition applications. For example, Tang et al. [7] achieved an average accuracy of 90.5% in classifying six sleeping postures using CNNs. However, CNNs are computationally intensive and may not be suitable for small mobile systems. In contrast, the pressure mat offers a simpler data format compared to images conventionally used in cognition tasks, facilitating online processing and learning with lightweight models.

While recent techniques based on deep neural networks have demonstrated promising accuracy [7,18], NN-based methods require large amounts of computation and memory, which cannot be implemented in resource-constrained devices like IoT devices. Commercially available pressure sensing mats are concerned with determining the presence of a person or object rather than detecting various activities and require costly high-resolution computation [20,21].

Also, conventional AI techniques are generally prone to noise in different components of the system [22]. The noise can stem from various sources, including sensor and input data fluctuations, communication interference between the sensors and the hardware, and memory errors [19,23]. Therefore, there is a need to design a pressure sensing system that is robust to noise while maintaining effectiveness (e.g., cost, time, and energy, to align with IoT device requirements) and ease of setup.

In this work, we propose a novel hyperdimensional computing (HDC)-based pressure mat design that outperforms the accuracy of state-of-the-art methods with clean data (without noise) as well as in the presence of different types of noise. HDC is an ideal lightweight computing model inspired by the cognitive process of the brain [24,25]. Our proposed design improves the traditional HDC by including a novel encoding mechanism, and provides a low-cost, time- and energy-efficient, and robust pressure sensing system for smart home applications. Our HDC-based model demonstrates resilience across a spectrum of noise sources, spanning from initial data acquisition to wireless transmission, with the overarching goal of mitigating the impact of noise on overall system performance. By uniformly distributing the information over long vectors, HDC is well-suited to handle such noisy data effectively [26]. Also, benefiting from simple arithmetic computations, HDC can be highly efficient in terms of computation time and energy, making it very suitable for IoT environments [27,28]. This is the first paper that investigates an HDC-based method on pressure mat data by introducing a novel encoding approach that is robust to multiple sources of noise.

To summarize, our work makes the following key contributions:

- We designed a low-cost pressure sensing mat with 512 sensing areas using Velostat material [29] for indoor activity recognition.
- To address the computational constraints of such a small mobile system, we developed a novel lightweight online learning algorithm based on noise-resilient HDC. To the best of our knowledge, this paper represents the first application of an HDC-based method for the analysis of pressure mat data. We enhance traditional HDC performance with a novel encoding method to account for different noise sources, all while ensuring comparable time and computation costs by operating in significantly lower dimensions (vector lengths).
- To evaluate the performance of our system, we collected two datasets, capturing the static and continuous nature of human movements. Our study includes three static and four continuous activities. Our system matches CNN’s accuracy for static activities and outperforms it by 9.47% for continuous activities with an accuracy of 93.19%, while consuming 85% less energy. This is achieved with compact 200-dimensional vectors, compared to the more memory-intensive HDC baseline (2*k*–10*k* vector dimensions).
- Our method effectively preserves complex data patterns without requiring complicated feature extraction, operating directly on raw data. Extensive experiments, including noise injections, demonstrate its superiority in smart home activity recognition over state-of-the-art algorithms. Our approach excels in mitigating noise challenges, achieving an average improvement of 13.19% (8.77%) for continuous (static) data with Gaussian noise compared to the current state-of-the-art method.

The rest of the paper is organized as follows: Section 2 and Section 3, respectively, provide the background on related works and on the hyperdimensional computing background. Section 4 explains our proposed hardware and software design. Section 5 describes the experimental setup and presents the results. The paper concludes in Section 6.

## 2. Related Work

Existing works on human activity recognition using pressure mats can be divided into three categories according to their specific features as follows: system design, hardware design, and algorithm design. Ref. [7] utilized a CNN for posture recognition and achieved a high accuracy of around 90%. The study also involved the use of real-time feedback, posture diaries, and fall alerts through a smartphone application, offering an advanced and accessible solution compared to intrusive and expensive alternatives for clinical sleep monitoring. Ref. [17] introduced a novel contactless sleep-monitoring IoT system with RFID-tagged bed sheets and a Raspberry Pi 4 Model B, addressing the limitations of conventional sleep posture monitoring based on the Random Forest approach. Ref. [19] introduced PressurePose, a synthetic dataset featuring a large pressure images dataset with 3D human poses and shapes, created through a physics-based simulation method. The accompanying PressureNet is a deep learning model that, with the aid of a pressure map reconstruction (PMR) network, estimates human pose and shape from pressure images, exhibiting robust performance with real data of diverse poses.

Traditional neural network (NN)-based methods, as seen in studies like [7,17], face challenges due to their reliance on large datasets and numerous parameters. Existing approaches often involve computation- and energy-intensive algorithms, such as feature extraction, which are impractical for resource-constrained devices like IoT devices. Additionally, these methods are susceptible to noise in various regions of their systems.

In our work, we introduce system, hardware, and algorithm designs. Our paper represents the first investigation of an HDC-based method applied to pressure mat data, incorporating a novel encoding approach. The proposed method operates directly on raw data, eliminating the need for complex computations, like feature extraction, parameter-tuning, and the time- and energy-consuming processes typical in NN-based methods. Hence, it can be easily implemented in resource-constrained devices like IoT devices. Our proposed method not only outperforms state-of-the-art accuracy levels under clean data conditions but also demonstrates robust performance in the presence of different kinds of noise, owing to its ability to capture local patterns in the images. Furthermore, this is the first study that analyzes the memory and communication, input, and sensor noise data commonly observed in human activity recognition systems and yields considerably higher accuracy compared to the best state-of-the-art methods. We further summarize related works in Table 1.

## 3. Hyperdimensional Computing

Hyperdimensional computing (HDC) is a computational paradigm rooted in the principles of cognitive neuroscience, which is designed to process and represent information within high-dimensional spaces [24]. The key idea behind HDC is to leverage the inherent properties of high-dimensional spaces, such as redundancy and fault-tolerance, to enable robust information processing and representation. In HDC, the data are represented as high-dimensional vectors, which are manipulated using simple element-wise logical operations, such as AND, OR, and XOR, to perform computations.

**Figure 1:**
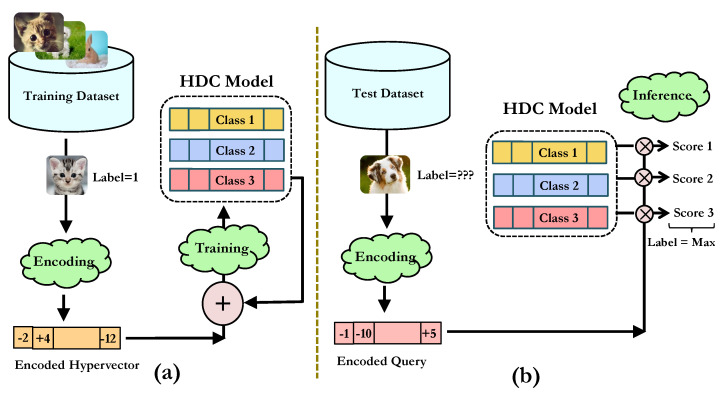
(**a**) HDC training. Each training data point is encoded to a hypervector and added up to the proper class hypervector based on its label. (**b**) HDC inference. The query data are encoded and compared with all the class hypervectors. The class index with the highest similarity is the prediction result.

HDC exhibits inherent noise tolerance due to the redundancy of the high-dimensional representation, allowing for robust recognition in the presence of noise in high-dimensional space [22]. We discuss the general steps for deploying a conventional HDC for a classification task. Figure 1 depicts the HDC steps. First, the input data are encoded into *D*-dimensional hypervectors. For encoded vectors ${\vec{H}}_{1}$ and ${\vec{H}}_{2}$, the proximity of $\vec{S}={\vec{H}}_{1}+{\vec{H}}_{2}$ to ${\vec{H}}_{1}$ and ${\vec{H}}_{2}$ is higher than to any other random vector. HDC algorithms capitalize on this principle to represent sets by aggregating together vectors of identical labels. After encoding, hypervectors of the same class are superimposed (added) to create *class* hypervectors. Then, the query data are classified using similarity metrics with respect to the *class* hypervectors, such as the cosine distance or the dot-product [25]. Later in Section 4.1, we introduce an innovative HDC-based algorithm crafted to augment the baseline HDC performance and to enhance its resilience towards various forms of noise.

**Encoding**: The most critical part of the HDC framework is the encoding process, which transforms the raw data into high-dimensional vectors. This is typically achieved by selecting a fixed set of random binary or bipolar vectors, and associating each unique input item or feature with one of these hypervectors. The association can be performed using different encoding methods, like random projection. An input sample is represented by a vector $F=\langle f_{1},f_{2},\cdots ,f_{d}\rangle$, where each element $f_{i}\in R$ corresponds to a feature, such as pixel values in the case of image representation. Table 2 summarizes the notations used throughout the paper.

Applying the framework of the random projection (RP) encoding technique, which is a popular encoding method [28], we assign a constant *D*-dimensional projection vector ${\vec{B}}_{i}$ to each feature position, resulting in a total of *d* constant vectors. The formulation of the random projection encoding can be expressed as follows:

(1) $$\vec{H}=\mathrm{sign}(\sum_{i=1}^{d}f_{i}\times {\vec{B}}_{i})=\mathrm{sign}\left(B\times F\right)$$

**Training**: During training, the encoded binary vectors are superimposed to form composite vectors representing each class. The superposition operation aggregates the encoded vectors belonging to the same class by element-wise addition, resulting in composite vectors that capture the shared characteristics and common features of the class. This process forms class-specific vectors that represent the aggregated information from the training samples. By superimposing vectors within classes, HDC leverages the cumulative knowledge to create robust and discriminative representations. The resulting set of class hypervectors serves as a reference for subsequent classification and inference tasks, enabling the recognition of patterns and concepts in new, unseen data. During the training phase, for every training sample $F^{j}$ with a corresponding label $y_{j}=\ell$, the HDC algorithm generates and accumulates encoded vectors ${\vec{H}}^{j}$ to form the class vector ${\vec{C}}^{\ell }$. This aggregation is achieved through the following equation:

(2) $${\vec{C}}^{\ell }=\sum_{j\;\mathrm{such}\;\mathrm{that}\;y_{j}=\ell }{\vec{H}}^{j}$$

Here, the class vector ${\vec{C}}^{\ell }$ for class label *ℓ* is formed by summing up the encoded vectors ${\vec{H}}^{j}$ of all the training samples with label $y_{j}=\ell$.

**Inference**: This step in HDC involves computing the similarity between an input data sample and the class hypervectors. This is achieved using element-wise operations between the encoded input $\vec{H}$ and the class hypervectors, which results in a binary vector that indicates the degree of similarity between the input and each class. The classification of the input is then determined based on the closest class hypervector as follows:

(3) $$\ell^{★}=\underset{j\in C}{\mathrm{argmax}\;}\left(\vec{H}\cdot \vec{C^{j}}\right)$$

**Retraining**: To enhance the accuracy, the retraining phase performs inference on the training samples and updates the class vectors. This involves adding the mis-predicted vector $\vec{H}$ to the correct class vector ${\vec{C}}^{\ell }$, while subtracting it from the erroneously assigned class vector ${\vec{C}}^{\ell^{\prime }}$. The update process can be represented by the following equations:

(4) $${\vec{C}}^{\ell }={\vec{C}}^{\ell }+\vec{H}\;\;{\vec{C}}^{\ell^{\prime }}={\vec{C}}^{\ell^{\prime }}-\vec{H}$$

While the baseline HDC excels in lower time and energy consumption compared to the NN-based methods [7,17], it faces challenges in detecting patterns in image data. Our enhanced HDC adopts 200-dimensional vectors, achieving superior efficiency. This enhancement ensures prolonged device lifespan, reduced operational costs, and enhanced sustainability. While showing notable noise resilience, in the noise-free scenarios, our proposed method also obtains a higher accuracy as it better stores the local patterns within the images.

### Hardware Design: Sensor Mat Design for Smart Home Pressure Sensing

**Figure 2:**
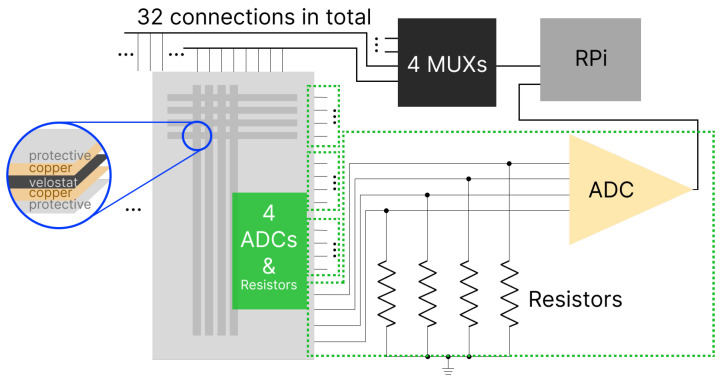
Conceptual diagram for the hardware design including a pressure mat, multiplexers (MUX), analog–digital-converters (ADC), and a Raspberry Pi (RPi) for data processing and learning.

The hardware of our system has three major components as follows: a sensor mat, multiplexers (MUX), and analog to digital converters (ADC). A conceptual diagram for our hardware is shown in Figure 2 (a picture of the practical setup is later shown in Figure 9).

## 4. Noise-Resilient HDC-Based Pressure Mat Design

Our pressure mat system is designed to work for both static and continuous activities. Static postures encompass no_press (no movement on the mat), standing still, and object containing, while continuous activities account for jump, walk, left/right (lr)_shift, and tiptoe. For all activities, we collect time-series data over multiple frames and label them manually. Our goal is the accurate prediction of activity types when presented with new samples. By leveraging our collected data and employing robust prediction algorithms, we strive to achieve precise recognition of various activities in real-time scenarios. Our smart sensing system comprises two integral components: (i) a custom-designed pressure mat hardware equipped with essential signal processing capabilities, and (ii) a novel lightweight online learning algorithm software based on HDC.

The hardware component captures analog signals generated by human body movement on the pressure mat, converting them into digital data records. This conversion process ensures compatibility and facilitates subsequent analysis. Each input dataset, regardless of activity classification, consists of 32 × 16 × 7 raw values representing a 7-frame time series. The software component, operating on a Raspberry Pi, receives input data with dimensions 32 × 16 × 7. These seven signals are treated as individual frames. The Raspberry Pi’s software is responsible for data preparation, online training, and generating the classification results. In the following, we explain the design of each component in more detail.

We chose the Velostat material [29] for its cost-effectiveness and established functionality as a force-sensitive resistor [30]. For signal management, four MAX4617CPE chips were selected as multiplexers (MUX) due to their proven reliability and performance in signal-switching applications. For the conversion of analog signals from Velostat to digital values, we employed four ADS1115 analog-to-digital converters (ADCs). This choice was based on the precision and suitability of ADS1115 for low-power applications [31]. Each ADC receives four voltage signals; thus, a total of 16 values are obtained from all four ADCs each time when one channel is open. We also carefully select resistors as part of the voltage divider to customize the voltage range.

Our design goal is to create a low-cost and high-resolution hardware solution tailored for daily smart-home scenarios. We achieve this by constructing a pressure-sensitive mat using Velostat [29], a polymeric foil impregnated with carbon black, effectively serving as a force-sensitive resistor. In comparison to traditional force-sensitive resistors, Velostat offers a cost-effective alternative with equivalent functionality for smart-home applications [30]. We meticulously affix 0.5-inch carbon tapes onto the Velostat mat, delineating distinct sensing areas chosen by the multiplexers (MUXes) [32] and identified by the analog-to-digital converters (ADCs) [33]. Each intersection of tapes creates a 0.25 square inch sensing area. Having 32 vertical and 16 horizontal tapes, we are able to create a low-cost and high-resolution pressure mat with 512 sensing areas. We also have four 8:3 MUXes to control the 32 signals. Only one signal is allowed for triggering high voltage at a time. This operation could avoid high voltage aliasing. Since we have all the 32 lines sharing with the load resistors, it is important to separate the high-voltage releases between them. The decisions are made based on common practice reported in the literature [30,34].

**Figure 3:**
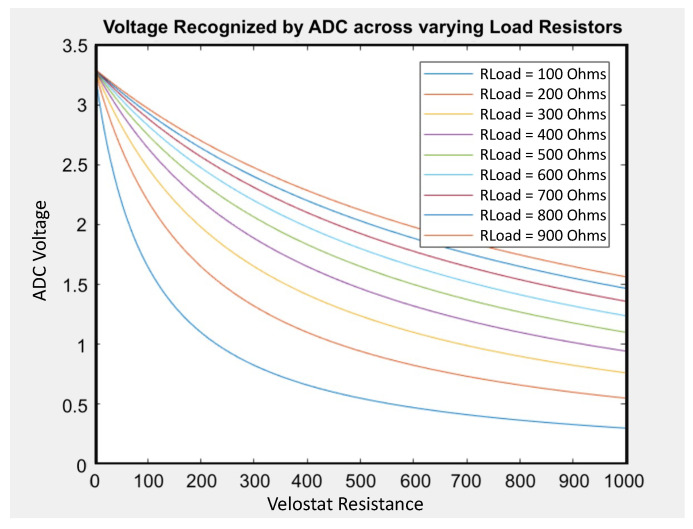
Voltage at ADC for load resistors in series with Velostat.

One issue with Velostat is its nonlinear voltage-resistance behavior. Figure 3 displays the voltage recognized by ADCs under various Velostat resistance and load resistors. Using a 100 Ω load resistor can generate 0.3–3.25 V output, but using 900 Ω results in only 1.6–3.25 V. In order to have a greater resolution, we use smaller resistors. However, as the resistance drops, the voltage–resistance curve becomes more nonlinear. This means that if the resistance decreases, nonlinear behavior increases. Although we prefer low resistance for high resolution, we need to keep the resistance value high enough for more linear outputs. Therefore, we chose 200 Ω as the middle value to balance the two trade-offs. Choosing the right load resistor is important to gain sufficient resolution as well as to reduce the negative impact of the nonlinear relationship.

### 4.1. Software Design: HDC-Based Classifier

**Data pre-processing**: To prepare for training, we begin by conducting data pre-processing. Each data frame consists of 32 × 16 digital values obtained from the ADC conversion. In the dataset, we differentiate between two cases: static postures and moving activities. For static postures, we combine identical 2D matrices from 7 frames of 32 × 16 into a single sample. On the other hand, for moving activities, we collect the matrices in chronological order to capture the temporal progression of the motion. We then perform separate training for the static and dynamic data.

**Figure 4:**
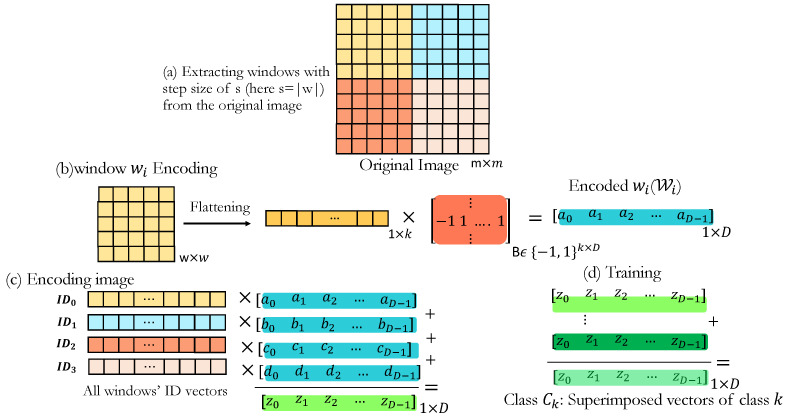
Proposed encoding: (**a**) Splitting original image to smaller windows, (**b**) encoding a singular window using conventional random projection encoding, (**c**) ID-based encoding of the original image, and (**d**) training.

**HDC-based Learning and Classification**: Figure 4 illustrates the proposed method’s flow for one frame input image. To better preserve the local information in different windows of images rather than merely considering an image holistically, we propose a novel window-based encoding method that effectively preserves the local features within the input data while mapping them to a hyperspace. The goal is to capture and retain the important temporal and spatial information without relying on complex feature extraction techniques.

Our approach leverages the idea that traditional HDC encoding does not explicitly preserve the temporal and spatial characteristics of the images. By encoding multiple windows within each image, we can retain a larger amount of relevant information from encoding local patterns. Moreover, the resulting hypervectors are more tolerant to noise due to the increased information preservation achieved through the window-based encoding process.

**Window-based Encoding (Local Encoding)**: Our window-based HDC encoding is inspired by the effective use of convolutional filters in extracting local features from images within convolutional neural networks, where nuanced local information is captured. In this technique, instead of considering the input image as a single image and encoding it, we consider fixed window sizes of w×w within an image of size m×m, and perform the encoding on each window separately (Figure 4b) using random projection encoding explained in Section 3. This ensures preserving the local patterns in smaller regions. The resulting hypervectors, denoted by $W_{i}$, are then superimposed to create the hypervector representing the overall image W (Figure 4c), as follows:

(5) $$\vec{W}=\sum_{i=1}^{n}{\vec{W}}_{i}\cdot ID_{i},$$

where *n* is the number of windows within an image. We bind (via multiplication) each encoded $W_{i}$ by a random *D*-dimensional ID vector to distinguish between different window locations [35]. An image of size m×m will have $n={\left(\frac{w(m-w)}{s}\right)}^{2}$ windows with an overlapping step size of *s*. The selection of *w* (window size) and overlapping step *s* is tentative and can be optimized based on the dataset. For our case, we obtained slightly better accuracy with w=8 and s=3. Window-based encoding is based on encoding different overlapping windows within an image, which allows us to capture the local and spatiotemporal patterns present in the data. It also provides the opportunity to perform the encodings of smaller windows in an image simultaneously, preserving the time and computation overhead.

**Dynamic data encoding**: After encoding each image frame as represented in Figure 4, we implement a similar approach for encoding a series of frames as in Figure 4c, where different frames are distinguished by allocating a unique $ID_{\mathrm{frame}\;i}$ to each frame *i*.

(6) $$W_{\mathrm{dynamic}}=\sum_{i=1}^{\#frames}{\vec{W}}_{\mathrm{frame}\;i}\cdot ID_{\mathrm{frame}\;i}$$

**Training and Inference**: We aggregate the encoded vectors that belong to each class to obtain a single class hypervector, as discussed for baseline HDC [25], representing each class. For prediction, we perform a similarity check between the unclassified testing hypervector $H_{test}$ encoded from the testing sample and all the trained class hypervectors. A basic cosine similarity check is used to find the closest hypervector whose class is the predicted class, i.e., $\arg\max_{j}\cos\left(H_{test},C^{j}\right)=\frac{H_{test}\cdot C^{j}}{\left|\right|H_{test}\left|\right|\cdot \left|\right|C^{j}\left|\right|}$. The class exhibiting the greater similarity to the encoded query is assigned as the label for the query data.

## 5. Experiments and Results

### 5.1. Experimental Setup

**Implementation**: We use a Raspberry Pi 4 for data collection and HDC-based learning. We implement a novel HDC-based encoding based on the *torch-hd* [36] package. Each training epoch will go through sequential neural networks with one HDC encoding layer. We chose to set *D* at 200 based on empirical observations, indicating that the encoded classes remain distinguishable even with relatively smaller vector sizes. This choice is supported by a thorough analysis of the accuracy and the capacity to be differentiated, as illustrated in the t-SNE plots in the following. *D* is set to 10,000 for the baseline HDC.

**Figure 5:**
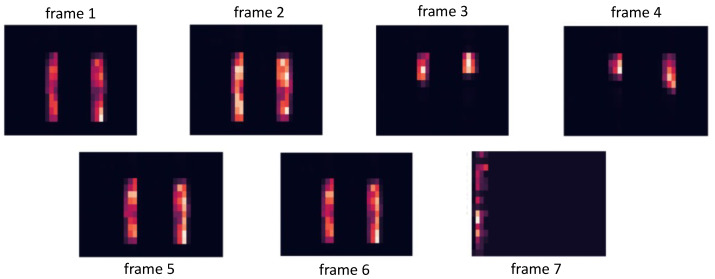
Visualization of the time-series tiptoe data.

**Dataset**: We construct a dataset with six different activities. Figure 9 illustrates the practical setup for capturing the images. Each set of input data, regardless of activity classification, consists of 32×16×7 raw values, representing a 7-frame time series. The software component later receives input data with dimensions 32×16×7 as individual frames. The Raspberry Pi’s software is responsible for the data preparation, online training, and generating the classification results. There are two static poses (no_press, stand) and four moving actions (jump, walk, lr_shift, and tiptoe). As an illustrative example, Figure 5 visualizes one sample of the tiptoe class that contains seven consecutive frames. As time moves forward, the frame changes from a full footprint to a half footprint with a tiptoe up, and finally lands back to the ground, which completes the full action of tiptoe movement including seven frames. Each static and dynamic set of data is separated into training and testing sets, using an 80–20% split.

**Baselines**: We employ a comprehensive set of baseline models for comparative evaluation, including convolutional neural network (CNN) [7], multi-layer perceptron (MLP) [17], support vector machine (SVM) [37], logistic regression (LR) [38], and a baseline random projection HDC [25]. The CNN architecture includes two convolutional layers with batch normalization, ReLU activations, and max-pooling, followed by two fully connected layers. Three ReLU activations culminate in a softmax activation function. The specific layer configurations are as follows: 1st convolutional layer (1 input channel, 32 output channels, kernel size 3, padding 1), 2nd convolutional layer (32 input channels, 64 output channels, kernel size 3, padding 1), and fully connected layers (256 neurons, ReLU activation, dropout *p* = 0.2, and 6 output neurons). The training spans 20 epochs with a batch size of 64, employing the Adam optimizer (lr = 0.0001) and cross-entropy loss. The results are averaged over 10 iterations. The MLP configuration involves a four-layer network and incorporates dropout layers, featuring hidden layer sizes of 128, 64, and 32, and an output size of 3 (4) for the classification task in static (dynamic) cases. The training process involves 20 epochs with a batch size of 32. In addition to two fully connected layers, the model incorporates rectified linear unit (ReLU) activations after each fully connected layer. The dropout layers are employed to enhance regularization during training. The final layer is activated using a softmax function. It is noteworthy that all the aforementioned parameters are extracted after an exhaustive hyperparameter tuning process to identify the optimal parameter configurations, ensuring a robust and fair comparison.

**Evaluation Metrics**: We evaluate the training performance for accuracy on a common test set at various epochs, execution times, and levels of energy consumption. We run 20 epochs for HDC (baseline [25] and our proposed design), CNN [7], and MLP [17]. All experiments are evaluated on RPi 4 [39]. The energy consumption is measured by a HIOKI 3334 power meter [40].

### 5.2. Accuracy, Execution Time, and Energy Consumption with No Noise

**Figure 6:**
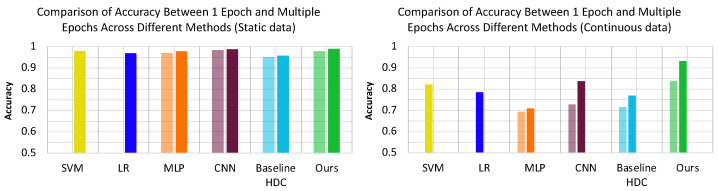
Accuracy comparison between 1 and 100 epochs of training across SVM [37], LR [38], MLP [17], CNN [7], Baseline HDC [25] and Proposed Method (static data on the left, dynamic data on the right).

**Accuracy**: Figure 6 compares the accuracy of the different state-of-the-art methods and baseline HDC [25] with our proposed method. We achieve comparable results as CNN (best state-of-the-art) on the static data, where CNN converges to 98.88% accuracy and HDC converges to 98.96% after fewer epochs. Continuous datasets are more challenging for classification tasks. Our HDC-based method achieves a significantly higher accuracy of 93.19% in contrast to CNN’s [7] (best state-of-the-art) 83.72%. More significantly, HDC achieves similar accuracy (83.84%) as multi-epoch CNN in only one epoch of training. In comparison to the baseline HDC [25], our proposed method yields 3.18% and 16.18% higher accuracy, respectively, for static and dynamic data.

**Figure 7:**
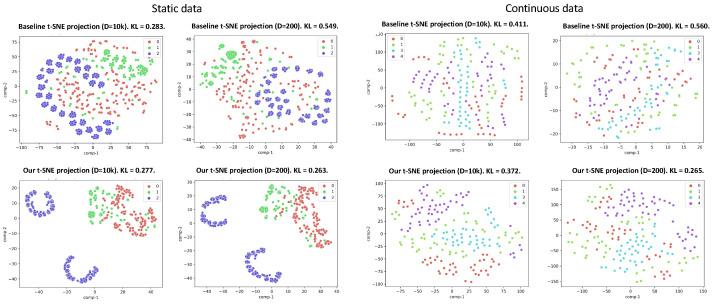
t-SNE plots illustrating the encoded hypervectors learned by our method for static data and continuous data compared to baseline HDC. The plots are generated with *D* = 10*k* and *D* = 200. KL denotes the Kullback–Leibler divergence. The bottom row is our method where the clusters are more distinguishable.

In Figure 7, we present a comparative analysis between our proposed method and the baseline random projection HDC [25]. Figure 7 displays t-SNE plots of the static and continuous data. The t-SNE plots visualize high-dimensional data by preserving the local similarities, revealing patterns in lower-dimensional space. The clusters display noticeable overlapping and outliers for both small and large hypervector dimensionalities (*D* = 200, 10*k*). However, the bottom plots, representing t-SNE plots for our proposed encodings, show more distinguishable clusters for both large and small dimensionality. The lower KL-divergence values further confirm the improved discriminative capability of our proposed method compared to the baseline HDC [25]. Specifically, for D=200, we obtain KL values of 0.264 (static) and 0.265 (dynamic)—notably lower than the baseline HDC [25], with 0.549 (0.560) for the static (dynamic) data. This trend persists with a higher dimension of *D* = 10*k*.

**Figure 8:**
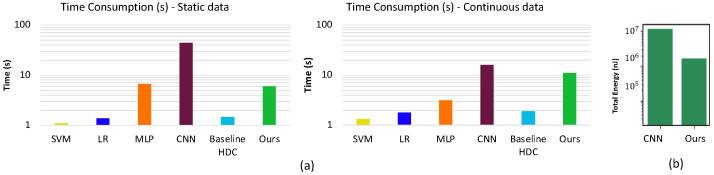
(**a**) Comparison of execution time of static data and dynamic data. (**b**) Shows average energy consumption comparison across SVM [37], LR [38], MLP [17], CNN [7], Baseline HDC [25] and Proposed Method.

**Execution Time**: The measured training and test execution time for the proposed HDC on RPi is shown in Figure 8. To reach comparable testing accuracy, the proposed method uses about 7.32×(1.44×) less total time than CNNs for static (dynamic) datasets, which demonstrates the efficiency of our design. In comparison to the other methods, our proposed method consumes higher energy but the overhead is not comparable with that of CNN [7].

**Energy consumption**: The measured energy on RPi is shown in Figure 8. To reach comparable testing accuracy, HDC uses about 85% less total energy than CNN, which demonstrates the efficiency of our design. HDC has similar power consumption per unit time to CNN, but CNN takes a much longer time to perform prediction and, therefore, uses more energy in total.

### 5.3. Accuracy with Noise

**Figure 9:**
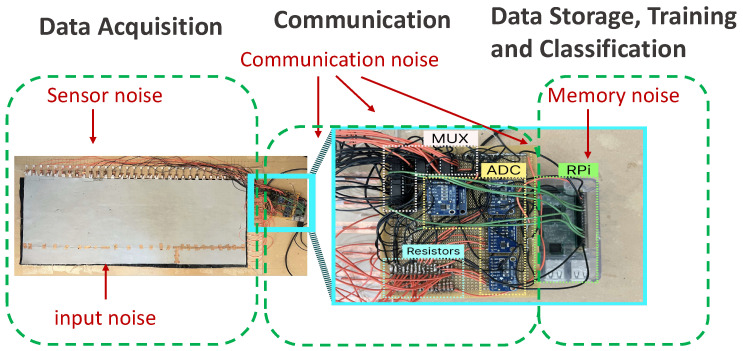
Different kinds of noise in our pressure mat system prototype: sensor, Input, communication, and memory noise.

In the operation of pressure mat systems, the acquired data are subject to various sources of noise inherent to these devices, originating from different components of the system. We systematically analyze the influence of different noise types on our system and compare the performance of our proposed method with the state-of-the-art approaches in terms of accuracy. We also report the execution time and the energy consumption. Based on our experiments, we observe that a lower reduction in accuracy corresponds to higher resilience towards noise. Figure 9 illustrates the specific locations within our system where each form of noise occurs. The examined noises include (i) input noise, (ii) sensor noise, and (iii) memory and communication noise, each of which will be explained and thoroughly investigated in the following sections. It is important to note that our experiments focus primarily on static data, as the introduction of noise in dynamic data may result in various combinations across frames, affecting either individual frames or the entirety of the dataset, which can be studied in future work.

#### 5.3.1. Input Noise

**Figure 10:**
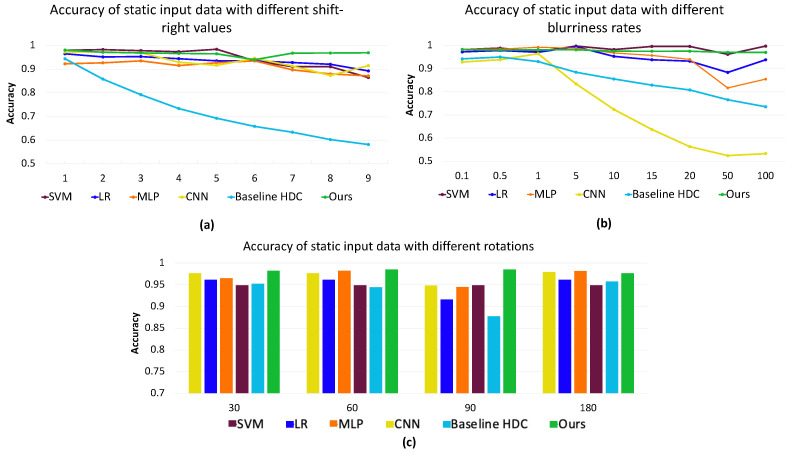
Effect of input noise, (**a**) shift-right, (**b**) blurriness, and (**c**) rotation, on the accuracy across SVM [37], LR [38], MLP [17], CNN [7], Baseline HDC [25] and Proposed Method for static data.

To evaluate the impact of input noise, we introduce variations simulating real-world scenarios, evaluating the resilience of our system to environmental influences and unexpected inputs. Understanding and addressing input noise is crucial for ensuring the accuracy and reliability of the data collected. Errors in the input data might occur during data acquisition, such as distortions in the data or losing a portion of the mat’s data. In our simulations, we introduce such errors by implementing shift, blurriness, and rotations, as discussed by [19]. The results are depicted in Figure 10.

The observations in Figure 10a reveal that as the information loss intensifies with further shifts, the accuracy of the system generally declines. This decline is particularly noticeable for the baseline HDC. Our proposed method consistently maintains high accuracy, outperforming the CNN [7] and baseline HDC [25] by, respectively, an average of 3.22% and 24.4% across shifts within the [1–9] range. Also, the accuracy of our method is, respectively, 1.37%, 3.21% and 1.22% higher than MLP [17], LR [38], and SVM [37]. For the blurriness noise, our proposed method yields a similar accuracy with SVM when different rates of noise are applied. Note that SVM and our proposed method are the best-performing methods when dealing with blurriness in the data. Also, when rotating, our proposed method outperforms the other methods by an average of 4.95%, 3.33%, and 1.21%, respectively, compared to baseline HDC [25], CNN [7], and SVM [37].

#### 5.3.2. Sensor Noise

**Figure 11:**
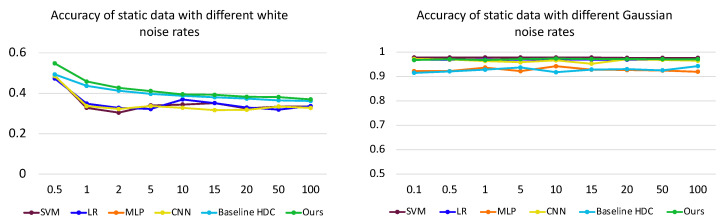
Effect of sensor noise (white and Gaussian on input data) on the accuracy for static data across SVM [37], LR [38], MLP [17], CNN [7], Baseline HDC [25] and Proposed Method.

We investigate the influence of sensor noises, specifically Gaussian noise, as discussed in [41], and white noise on the input data. We depict the accuracy change with different standard deviations, evaluating the performance of various methods on the static data. We depict the results in Figure 11. Our proposed method’s decline is lower than that of the other methods. To see how tolerant different methods are towards different kinds of noise types, we applied a range of noise intensities (see Figure 11). Our method maintains higher averages of 7.33% and 0.65%, respectively, with white and Gaussian noise within the different noise rates. Compared to the baseline HDC, these values are 1.7% and 4.23% higher.

#### 5.3.3. Memory and Communication Noise

As systems grow more complex, preserving data integrity faces increasing challenges during memory and communication processes. Our investigation thoroughly assesses the system’s resilience to memory corruption and communication disturbances, with a focus on maintaining stability and precision in the stored and transmitted information. We specifically address memory and communication noise, examining the impact of packet loss, Gaussian noise, and random bitflips within our system, as outlined in [23,42]. The literature highlights that Gaussian noise and random bitflips can occur in both memory and communication processes due to factors such as hardware limitations, electromagnetic interference, and software vulnerabilities [23,43].

To perform the analysis, we introduce each of packet loss, bitflips, and Gaussian noise—common occurrences in IoT networks—into our models. Specifically, for CNN [7] and MLP [17], we apply these noises to the model weights. In the case of HDC, which lacks model weights in the traditional sense, we explore the effects of injecting set-to-zero, bitflip, and Gaussian noise into the stored class hypervectors post-training and the encoded hypervectors. However, for bitflips, we exclusively consider the encoded hypervectors, as the class values are represented as floats. We note that, according to [44,45], the discussed sensor noise (Gaussian and white noise) could also be considered as forms of communication noise.

**Figure 12:**
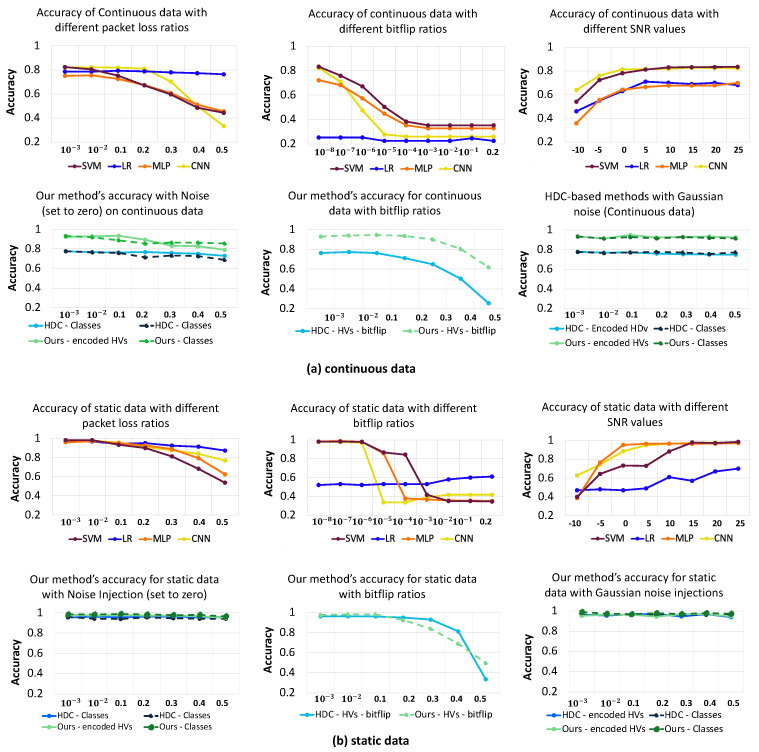
Effect of memory and communication noise on accuracy across SVM [37], LR [38], MLP [17], CNN [7], Baseline HDC [25] and Proposed Method for continuous data (**a**) and static data (**b**). The left, middle, and right columns show the results of packet loss, bitflip, and Gaussian noise, respectively.

Figure 12a,b illustrate the accuracy variations with different noise ratios for the continuous and static data across various methods. Our observations indicate that the baseline HDC model exhibits superior tolerance to set-to-zero (considered on a par with packet loss), bitflip, and Gaussian noise compared to the other methods for both continuous and static data. Notably, our proposed method surpasses the baseline HDC significantly. It demonstrates remarkable resilience to memory and communication noises, tolerating substantial ratios, whereas alternative methods experience a rapid decline in accuracy with even minor injected noise. More specifically, our proposed method outperforms the best state-of-the-art method (LR [38]) in packet loss noise resilience by an average of 19.9% (4.61%) for dynamic (static) data across different ratios of noise. Also, our proposed method yields up to an average of 33.77% (18.72%) higher accuracy for dynamic (static) data compared to the best state-of-the-art method (CNN [7]) in the presence of bitflip, and up to an average of 13.19% (8.77%) in the presence of Gaussian noise.

### 5.4. Discussion and Future Work

Unlike conventional machine learning (ML) and NN-based computing methods, HDC maps the input data into hypervectors with many elements, e.g., of the order of 2–10*k* elements. Our proposed method yields a high accuracy with vectors of as few as 200 dimensions. However, for more complex tasks and data, in order to achieve high accuracy, higher order dimensions are required. In such settings, if memory is limited and not optimized, storing large hypervectors can be challenging. Additionally, computing with high-dimensional vectors may become a potential bottleneck, leading to suboptimal performance and increased computational overhead. Furthermore, due to the predominantly linear nature of HDC encoding, it has limitations in capturing and classifying complex images [46]. While our proposed method improves the accuracy of the baseline HDC by preserving local patterns in images, advancing HDC encoding may be necessary when dealing with more complex data, such as combining NN-based feature extraction with HDC. This, however, goes beyond the current scope of HDC, which excels in robustness to noise, simplicity of operations, and lower computation and energy consumption.

In future work, we plan to advance our current methodology by harnessing the rich potential inherent in diverse data types, such as incorporating signals derived from human pose and shape analysis applied to pressure images. This enhancement is aimed at achieving more nuanced and accurate human activity recognition while remaining within the scope of HDC and using its simplicity, robustness, and resilience towards noise. Also, the work can be extended to incorporate classification of more complex cognition tasks based on heterogeneous data from different inputs. Additionally, we intend to conduct a comprehensive examination of datasets characterized by imbalanced labels and to study the impact of data augmentation on the performance of the HDC model. Further study also needs to be undertaken in terms of system maintenance, specifically in the presence of noise, as well using hyperspace sparsification techniques in HDC to utilize less memory.

## 6. Conclusions

In this study, we introduced a novel pressure sensing system, specifically designed for cost-effective and noise resilient human activity recognition. Our system includes a pressure mat sensor based on Velostat and a novel HDC classifier, which excels in lightweight computation and demonstrates resilience to various types of noise. We comprehensively evaluated our proposed system on a Raspberry Pi, demonstrating a remarkable 9.47% improvement in accuracy compared to the current state-of-the-art method (CNN) for dynamic data. Notably, our system achieves this superior accuracy while consuming 85% less energy and exhibiting a remarkable speed enhancement of up to 7.3× compared to existing methods. Furthermore, we confirm the robustness of our method in handling various noise types, such as memory and communication, sensor, and input noise. In the presence of communication and memory noise, our system enhances the final accuracy by up to 33.77% and 18.72% for dynamic and static data, respectively. When exposed to sensor noise, the accuracy is boosted by up to 7.33% and 0.63%, utilizing dynamic and static data. Additionally, it yields up to 3.33% higher accuracy in the presence of input noise compared to the best state-of-the-art method. Compared to the baseline HDC [25], our proposed method achieves significant accuracy improvements of up to 20% (4.23%) for dynamic (static) data in the presence of communication and memory noise. These findings emphasize the practical viability and efficiency of our proposed pressure sensing system, positioning it as a promising solution for applications requiring accurate and resource-efficient human activity detection.

## Figures and Tables

**Table 1:** Comparison of human activity recognition using pressure mat approaches.

Authors	System	Comparison
Tang et al. [7]	CNN-based posture recognition	Data and computing intensive, prone to noise
Chen et al. [17]	Raspberry Pi 4 and Random Forest	Data and computing intensive, prone to noise
Clever et al. [19]	Synthetic dataset (PressurePose)	Computing and energy intensive
Our Work	Novel HDC-based encoding design	Outperforms in noise resilience, time and energy efficient

**Table 2:** Table of notations.

Symbol	Definition	Symbol	Definition
*d*	Dimension of the original data	*B*	Base matrix
*D*	Dimension of encoded data	H	Hypervector of encoded data
*w*	Window size	W	Encoded window
*s*	Window overlapping step	*C*	Class hypervector

## Data Availability

The data presented in this study are available on request from the corresponding author.
